# Supplementary material for: AffyMAPSDetector: a software tool to characterize Affymetrix GeneChip™ expression arrays with respect to SNPs
Source: BMC Bioinformatics. 2007 Jul 30;8:276. doi: 10.1186/1471-2105-8-276 (PMC1959249; doi:10.1186/1471-2105-8-276)
Supplement: Additional file 13 — AffyMAPSDetector v1 source code. AffyMAPSDetector source code. [file 1471-2105-8-276-S13.zip › AffyMAPSDetector_v1_src/doc/affychip/snpdetector/util/GuiUtils.html]

GuiUtils


|  |  |  |  |
| --- | --- | --- | --- |
| **Overview** \| **Package** \| **Class** \| **Use** \| **Tree** \| **Deprecated** \| **Index** \| **Help** | | |  |
| **PREV CLASS**   **NEXT CLASS** | **FRAMES**    **NO FRAMES**     **All Classes** |  |  |
| SUMMARY: NESTED \| FIELD \| CONSTR \| METHOD | DETAIL: FIELD \| CONSTR \| METHOD |  |  |


---


## affychip.snpdetector.util Class GuiUtils

```
java.lang.Object
  affychip.snpdetector.util.GuiUtils
```

---

public class **GuiUtils** extends java.lang.Object

Title: Probe SNP Detector

Description: Affymetrix Gene-Probe SNP Detection User Interface Utility
Class.

Copyright: Sunita Kumari -- Copyright (c) 2004

Company: George Mason University

**Version:**
:   1.1

**Author:**
:   Sunita Kumari

---

**Constructor Summary**

`GuiUtils()`


|  |  |
| --- | --- |
| **Method Summary** | |
| `static void` | `scrollToCenter(javax.swing.JTable table, int rowIndex, int vColIndex)` |
| `static void` | `setFrameLocation(java.awt.Window frame, double horizontalPercent, double verticalPercent)`             Use this method if you want to place a `Window` relative to the top-left corner of graphical device screen. |

**Methods inherited from class java.lang.Object**

`clone, equals, finalize, getClass, hashCode, notify, notifyAll, toString, wait, wait, wait`

**Constructor Detail**

### GuiUtils

```
public GuiUtils()
```


**Method Detail**

### scrollToCenter

```
public static void scrollToCenter(javax.swing.JTable table,
                                  int rowIndex,
                                  int vColIndex)
```

---


### setFrameLocation

```
public static void setFrameLocation(java.awt.Window frame,
                                    double horizontalPercent,
                                    double verticalPercent)
```

:   Use this method if you want to place a `Window` relative to
    the top-left corner of graphical device screen.

    :   **Parameters:**: `frame` - type java.awt.Window -- The `Window` of interest to be placed on screen with respect to screen's top-left corner.: `horizontalPercent` - type double -- percentage/fraction of the screen-width to be used as X-coordinate of "frame" location.: `verticalPercent` - type double -- percentage/fraction of the screen-height to be used as Y-coordinate of "frame" location.


---


|  |  |  |  |
| --- | --- | --- | --- |
| **Overview** \| **Package** \| **Class** \| **Use** \| **Tree** \| **Deprecated** \| **Index** \| **Help** | | |  |
| **PREV CLASS**   **NEXT CLASS** | **FRAMES**    **NO FRAMES**     **All Classes** |  |  |
| SUMMARY: NESTED \| FIELD \| CONSTR \| METHOD | DETAIL: FIELD \| CONSTR \| METHOD |  |  |


---
